# Editorial: Trust: The Limits of Human Moral

**DOI:** 10.3389/fpsyg.2017.00178

**Published:** 2017-02-10

**Authors:** Panagiotis Mitkidis, Michaela Porubanova, Andreas Roepstorff

**Affiliations:** ^1^Department of Management, School of Business and Social Science, Aarhus UniversityAarhus, Denmark; ^2^Center for Advanced Hindsight, Social Science Research Institute, Duke UniversityDurham, NC, USA; ^3^Department of Psychology, Farmingdale State CollegeNew York, NY, USA; ^4^HUME Lab, Masaryk UniversityBrno, Czechia; ^5^New School for Social ResearchNew York, NY, USA; ^6^Interacting Minds Centre, Aarhus UniversityAarhus, Denmark; ^7^Department of Clinical Medicine, Center for Functionally Integrative Neuroscience, Aarhus UniversityAarhus, Denmark

**Keywords:** trust, cooperation, expectations, interaction, cognition, morality

The role of trust in human interaction has been a long-standing question in social sciences, and the interest has proliferated over the last few decades (Gambetta, 2000; Fukuyama, 1995; Dirks and Ferrin, 2001; Fehr, 2009). The majority of scientists agree that trust is a necessary ingredient for almost all functioning human interactions, from love and friendship to economic prosperity and the emergence of large-scale organizations (Slovic, 1993; Mayer et al., 1995; Fehr and Gächter, 1998; Hetherington, 1998; Fehr and Rockenbach, 2003). Research in psychology, anthropology, neuroscience, and economics has made tremendous advancements in identifying the psychological factors that promote trust behavior among humans (Adolphs, 2002; Delgado et al., 2005; King-Casas et al., 2005; Kosfeld et al., 2005; Williams and Bargh, 2008; Sellaro et al., 2014; Mitkidis et al., 2015). These findings have inspired the development of interventions that promote effective interactions and discourage free-riding (Ba et al., 2003; Ariely, 2009, 2016).

Trust is a public good and is based on social intelligence; “…a kind of intelligence that allows individuals to assess the degree of risk they may face in social situations when confronted with the possibility of interacting with strangers who might be the path to new and beneficial outcomes.” (Cook, 2001, xiii). Based on the above, it is obvious that trust is a risky endeavor given the uncertainty one may face when interacting with others^1^.

The first section of this special topic issue aspires to examine how people actually behave in situations of trust and to highlight the psychological and situational factors that foster trust. The second section aims to unravel some of the socio-cognitive and evolutionary mechanisms underlying trust and morality.

## How human interaction improves or decreases trust

How we can create and maintain positive human interaction has been both a historical question of the social sciences and a rapidly growing research topic (Lewis and Weigert, 1985; Rand et al., 2009; Wallot et al., 2016). Given how beneficial trust is in human relationships, it seems relevant to examine the factors that may increase or decrease trusting behavior and subsequently enhance cooperation. Trust motivation has been investigated by numerous studies (Yamagishi and Yamagishi, 1994; Kramer, 1999; Hardin, 2001; Mitkidis et al., 2013), illustrating that positive interactions with an individual or a group lead to higher trustworthiness and subsequently cooperation (Fehr and Fischbacher, 2004; Rand et al., 2009). In the real world, as well as in experiments involving social dilemmas, people do not always act in a purely selfish manner but rather display a variety of prosocial behaviors (Ostrom and Walker, 1991; Fehr and Gächter, 2000; Ariely, 2008). This raises the question: under which circumstances do we trust each other?

In this special issue on trust, several studies explored how the amount and the type of contact affects trusting behavior. Aarøe et al. demonstrate a negative association between individual's pathogen avoidance and social trust level. Håkonsson et al. examine both the selfish and trust behavior in organizations where communication is predominantly executed via different types of media. The authors generate hypotheses about how the media increase trust behavior in distributed teams. Michael et al. theorize that a necessary precursor to trusting behavior is a feeling of a sense of commitment. Andrighetto et al. investigate the factors other than fear of punishment, reputation, and others' expectations that can motivate trust. Their results propose that in the absence of the abovementioned incentives, normative expectations promote risky behaviors as trust is. Trust is therefore analogous to answering a very simple question: Can I trust you to do something? This question is a very practical one and reveals the essential mechanism behind interpersonal trust: the ability to predict actions of other people (Gambetta, 2000). Tackling this line of research, Hommel, and Colzato hypothesize that the degree of trust among individuals is largely determined by the degree of interpersonal similarity.

## Social cognition of trust

It has become widely accepted that people have cooperative preferences when taking into consideration the well-being of the group (Hamilton and Axelrod, 1981; Axelrod, 1984, 2006; Burton-Chellew and West, 2013; Rand and Nowak, 2013). Participating in cooperative tasks necessarily depends upon trust for the accomplishment of collective goals (Bratman, 1992; Cannon-Bowers and Salas, 2001). Empirically, trust has been associated with higher economic growth, the emergence of large-scale organizations, and lower levels of cheating in small-scale groups and corruption on the aggregate level of larger scale societies (Arrow, 1972; Putnam, 1993; Fukuyama, 1995; Knack and Keefer, 1997; Barr, 2003; Guiso et al., 2006; Johnson and Mislin, 2011). Contrary to the benefits of trust, its pitfalls are related to immoral behavior and potential corruption (Weisel and Shalvi, 2015).

Several works examine the link between trust and morality and the mechanisms underlying those behaviors. Collins et al. suggest that trust is a cognitive mechanism supplementary to other mechanisms within the cognitive system. Hochman et al. suggest that although people have an egoistic side, considerations that require some kind of social exchange and negotiation may dominate the self-interested instincts. Espín et al. argue that since people can stand on both sides of the trust game, namely act as both trustors and trustees, social preferences may considerably differ and extent from self-interest to efficiency and performance considerations. Tan et al. demonstrate that general system justification, a motivation to sustain the status quo of the society, is negatively associated with both corruption perception and corruption intention and that institutional trust plays a mediating role in these relations. Konis et al. provide insights into how a perception of immorality of an act differs for multiple victims in comparison to a single individual. Finally, Heintz et al. consider trust from an evolutionary perspective and hypothesize that human cognition evolved mainly for dealing with social life and despite its risky nature, trust may be advantageous.

## Conclusion: the limits of human moral

In the course of daily life, people routinely engage in social exchanges that involve some level of trust. Studies published as a part of the current research topic suggest that individuals do not automatically adopt trusting and moral behavior in human interactions, but that those behaviors are determined by and modulated by a plethora of cognitive, psychological, situational, and contextual factors. We believe that research published within the special topic significantly contributes to understanding of trust and we encourage further research both on the brighter and darker sides of trust.

## Author contributions

All authors listed, have made substantial, direct and intellectual contribution to the work, and approved it for publication.

### Conflict of interest statement

The authors declare that the research was conducted in the absence of any commercial or financial relationships that could be construed as a potential conflict of interest.

## Footnotes

^1^Maybe this is why Bob Dylan is so pessimistic when it comes to trust Dylan (1985).
